# Gain control of γ frequency activation by a novel feed forward disinhibitory loop: implications for normal and epileptic neural activity

**DOI:** 10.3389/fncir.2013.00183

**Published:** 2013-11-19

**Authors:** Zeinab Birjandian, Chakravarthi Narla, Michael O. Poulter

**Affiliations:** Department of Physiology and Pharmacology, Robarts Research Institute, University of Western OntarioLondon, ON, Canada

**Keywords:** epilepsy, disinhibition, interneuron, endopiriform cortex, olfactory cortex, piriform cortex

## Abstract

The inhibition of excitatory (pyramidal) neurons directly dampens their activity resulting in a suppression of neural network output. The inhibition of inhibitory cells is more complex. Inhibitory drive is known to gate neural network synchrony, but there is also a widely held view that it may augment excitability by reducing inhibitory cell activity, a process termed disinhibition. Surprisingly, however, disinhibition has never been demonstrated to be an important mechanism that augments or drives the activity of excitatory neurons in a functioning neural circuit. Using voltage sensitive dye imaging (VSDI) we show that 20–80 Hz stimulus trains, β–γ activation, of the olfactory cortex pyramidal cells in layer II leads to a subsequent reduction in inhibitory interneuron activity that augments the efficacy of the initial stimulus. This disinhibition occurs with a lag of about 150–250 ms after the initial excitation of the layer 2 pyramidal cell layer. In addition, activation of the endopiriform nucleus also arises just before the disinhibitory phase with a lag of about 40–80 ms. Preventing the spread of action potentials from layer II stopped the excitation of the endopiriform nucleus, abolished the disinhibitory activity, and reduced the excitation of layer II cells. After the induction of experimental epilepsy the disinhibition was more intense with a concomitant increase in excitatory cell activity. Our observations provide the first evidence of feed forward disinhibition loop that augments excitatory neurotransmission, a mechanism that could play an important role in the development of epileptic seizures.

## Introduction

Inhibitory synaptic transmission reduces the probability of action potential generation and synchronizes excitatory neuron activity thereby controlling brain neural network oscillations (Traub et al., 1999). There have been, in recent years, many studies that have shown a wide variety of interneurons that differ based on their activity, morphology, and neuropeptide expression. The role for this heterogeneity is thought to be important for gating of pyramidal cell firing in the CNS (Ascoli et al., 2008; Klausberger and Somogyi, 2008). There is also a widely held view that inhibitory activity can also increase the efficacy of an excitatory stimulus by reducing the activity of inhibitory cells that innervate excitatory cells, a process that is termed disinhibition. This has been shown to occur in the hippocampus (Toth et al., 1997), but few other examples have been documented also see (Xu et al., 2013).

Layer III of the olfactory or piriform cortex (PCtx) is composed almost entirely of a relatively high density of inhibitory interneurons that densely innervate the layer II principal cells (Gavrilovici et al., 2010). Recent studies have shown that differing inhibitory activity controls olfactory processing in the PCtx (Isaacson, 2010; Franks et al., 2011). However, how this innervation controls PCtx excitability and/or may be responsible to high seizure susceptibility of the PCtx is not clear. In particular, the anatomical and functional evidence showing this dense inhibitory innervation of principal cells is difficult to reconcile with the high epileptiform activity (Piredda and Gale, 1985; Demir et al., 1999, 2000, 2001). Therefore, the purpose of this study was to investigate the activation of the PCtx circuitry in order to see how layer III interneurons control normal and epileptic activity. To do this we used voltage sensitive dye imaging (VSDI) to record the electrical activity of acutely isolated brain slices that include the three layers of the PCtx as well as the underlying dorsal endopiriform nucleus (DEn). VSDI permits the visualization and study of relatively intact neural circuits both *in vitro* and *in vivo* (Carlson and Coulter, 2008; Coulter et al., 2011; McVea et al., 2012) and has been shown to faithfully indicate the activity of neural circuits in a wide range of brain areas. It has been particularly useful for the study of circuit behavior in pathophysiological conditions. For example (Ang et al., 2006) showed in an animal model of epilepsy that there is a wide spread loss of inhibitory control of hippocampal CA1 excitability. VSDI has also been used *in vivo* to show that after stroke the activity of entire mouse cerebral hemispheres can change in a manner that redistributes neural activity in an attempt to maintain normal functionality (Mohajerani et al., 2011).

We show that the activation of layer II principal cells cause the disinhibition of layer III interneurons that innervate layer II, thereby augmenting the initial excitatory drive and response. Furthermore, in an animal model of epilepsy we show that this feedback loop is strengthened. Thus, providing evidence of a previously unexplored mechanism by which epileptic seizures may arise.

## Materials and methods

All experiments were conducted in accordance with the guidelines of the Canadian Council on Animal Care and approved by University of Western Ontario council on animal care.

### Slice preparation and staining

All animals used in these studies were adult male Sprague-Dawley rats aged 20–45 days. The preparation of brain slices and kindling methodology have been described in detail elsewhere (Gavrilovici et al., 2006). Slices prepared from kindled rats were usually about 45 days old. Control rats for these experiments were age matched but no electrode was implanted. Brain slices were incubated for 30 min in a solution that contained 0.6 mM of dye di-4-ANEPPS (D-1199, Invitrogen Molecular Probes Inc., OR, USA). After washing for 10 min with ACSF slices were transferred to the recording chamber. During all recordings the slices were maintained at 32°C and continuously perfused with ACSF bubbled with a mixture of 95/5% oxygen and carbon dioxide. The slices were stimulated with a platinum/iridium electrode (MicroProbes, Inc., MD, USA) with a tip diameter of 200–300 μm at the border of the lateral olfactory tract (LOT) and layer I of the PCtx. The stimulation of each slice was in the range of 160–200 μA, each square pulse was 2.0 ms in length. The electrode was connected to a stimulator (S88X dual output square pulse stimulator, Grass Technologies, An Astro-Med, Inc., QC, Canada), which controlled the pulse frequency and train duration.

### Patch clamp recording

The whole cell patch clamp recording technique used and the preparation of brain slices from adult rats have both been described in detail elsewhere (McIntyre et al., 2002; Gavrilovici et al., 2012). The internal solution used in these experiments was in mM: K gluconate, 140; MgCl_2_, 2, CaCl_2,_, 1; MgATP, 2; NaGTP, 0.2; EGTA, 1.1 and HEPES, 10. A Multiclamp 700B amplifier was used to record from neurons located in layers II and III.

### Optical recording

**Figure 1:**
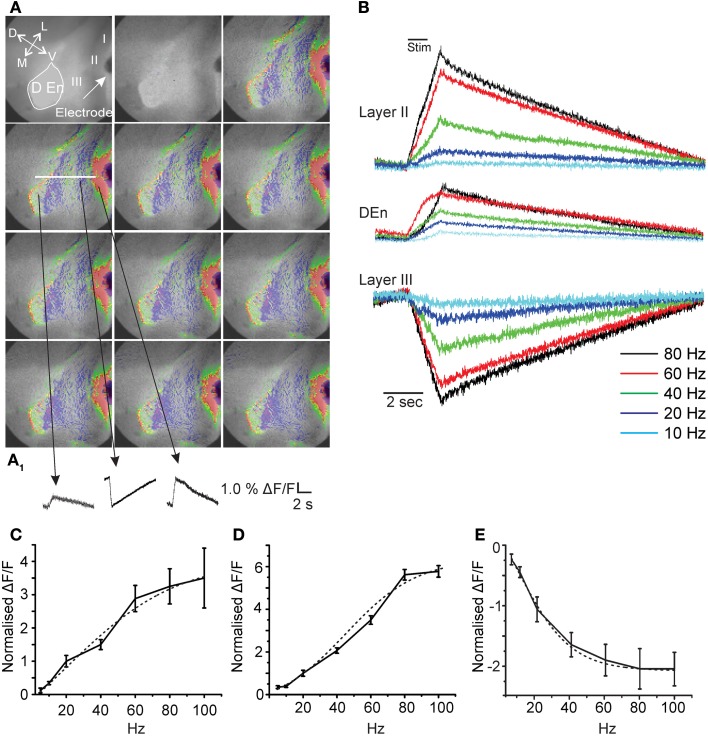
**Activation of the piriform cortex involves three kinds of responses originating in differing anatomical regions.** Panels in **(A)** show representative images from a 20 s long recording where a 40 Hz stimulation was used (red, green, yellow represents depolarization; blue magenta hyperpolarization). **(A_**1**_)** Activation causes depolarizing responses from layer II and the Dorsal endopiriform nucleus (DEn) and a hyperpolarizing response in Layer III. (arrow represents orientation of slice D: dorsal; V: ventral; L:lateral; M: medial). **(B)** Representative traces from one recording over the entire frequency range of stimulations used. Responses to stimulations less than 20 Hz were small and variable. **(C)** Shows two examples of whole cell patch recordings made form layer II or layer III neurons. Stimulation at 60 Hz for 1 s induced a large depolarization of pyramidal cells that generated action potential for 10–15 s after the stimulus. By contrast the same train did not excite cells in layer III but in contrast action potential generation was suppressed. **(D,E)** Graphs of normalized change in Δ F/F vs. stimulus intensity from 8 recordings in the layer II, Den, and Layer III, respectively. Activation of each response increased over the range of stimulation of 20–80 Hz (γ range). Note that 100 Hz stimulation was no more effective than 80 Hz. Dotted lines are fitted sigmoidal functions showing non-linearity for input/output relation.

The composition of ACSF used for optical recordings was the same composition used in the patch clamp recordings. Each recording was about 20 s in length and consisted of two époques. The first was a 2 s recording of background activity before the stimulus followed by the stimulus application for 1 s with frequencies differing from 5 to 100 Hz. The acquisition rate was between 3 and 10 ms/frame. For each recording minimum camera saturation was set around 50% while the maximum was about 80%. Optical recording was conducted using a CMOS camera (Micam Ultima, BrainVision, Inc., Tokyo, Japan) mounted on top of an upright microscope (Fixed Stage Upright Microscope BX51WI, Olympus). The light from a 100 W halogen lamp source (HLX 64625, Microlites Scientific, Corp.) passed through an excitation filter (λ = 530 ± 10 nm). The fluorescent signals were collected and projected onto the CMOS sensor through a long pass emission filter (λ > 590 nm). A long distance objective was used in these experiments (XLFluor 4X N.A. 0.28, Olympus). The movies were recorded and analyzed using Brain Vision Analyzer (Tokyo, Japan) software. The acquisition settings were: 100 × 100 pixels frame size, after magnification each represented 25 μm × 25 μm space on the brain slice. The dye signal intensity decreases as the membrane depolarizes. However, to better match conventional recordings the signals all have been converted so that the excitatory and inhibitory signals were shown as positive and negative values. As bleaching can strongly affect the data, all recordings were corrected by subtracting the change in fluorescence that occurred in a region of the slice that was unresponsive to the stimulus. The fractional change in fluorescence signal relative to background signal (ΔF/F) was calculated for each frame of each recording. For all the recordings, we binned 3 × 3 pixels into one representative signal. As there was considerable variability in the magnitude of the responses from slice to slice due to differences in loading of the dye, we normalized the recordings by dividing all signals by the response to the 20 Hz stimuli. This permitted us to average the normalized responses between recordings. Thus, the input/output relationships shown in Figures 1, 3–5 and 7 are the normalized Δ F/F. The lag time was calculated by measuring the time between the stimulus onset and the time for the signal to be 20% above baseline. For Figure 6, instead of using pixels bins, we measured the Δ F/F along a “stripe” that could be precisely placed along a group of pixels before and after the cut. Each stripe consisted of 10 pixels and covered 250 μm length. The data derived from each stripe was the average Δ F/F of 10 pixels.

Fitting of the input/output relationships in Figure 1 used a sigmoidal dose/response function run Origin 8.5.1 software package (North Hampton MA, USA).

To test how the VSDI signal follows the fluctuation of voltage in the cell membrane, we performed a number of control recordings where a high-K^+^ solution ([K^+^]^0^ = 80 mM) was perfused on the bath. Switching from control Ringer's solution ([K^+^]^0^ = 3 mM) to high-K^+^ solution should cause a depolarization in the membrane potential. The results showed that the fluorescence signal decreased, consistent with the depolarizing effect of the high-K^+^ solution. This switch caused, on average, a 6.3 ± 0.6% decrease in fluorescence. Assuming RMP was about −70 mV before perfusing the slice and the high-K^+^ ACSF depolarized the cells to 0 mV this corresponds to about 0.9% change in fluorescence for every 10 mV of membrane potential. We also confirmed in another set of control experiments that blocking action potential propagation using tetrodotoxin blocked all activity although a depolarization around the stimulation electrode was still evident.

### Statistics

Comparison of all measured values was done by either One or Two Way ANOVA as appropriate. The significance was set at *p* < 0.05. *Post-Hoc* comparisons were carried out using a Fisher Exact Test using Statview.

## Results

First, in order to characterize the activity of this preparation we stimulated the LOT using a bipolar electrode at differing frequencies for 1 s. We found that single pulses were ineffective at activating any prolonged circuit activity. However, at higher frequencies we found prolonged responses having both excitatory and inhibitory components. In Figure 1A we show a typical response to a 1 s train of pulses delivered at 40 Hz onto the LOT. The time between each image is about 2 s. Although, the response is heterogeneous three consistent aspects in all recordings were found. There were always two excitatory responses, one in layers I–II and another originating in the dorsal endopiriform nucleus (DEn; the first and third traces, respectively, in the bottom of Figure 1A). A third type of response indicated reduced activity in Layer III of the olfactory cortex. This layer contains little or no excitatory cells and is primarily composed of inhibitory interneurons that innervate the layer II pyramidal cells (Haberly et al., 1987; Behan and Haberly, 1999; Haberly, 2001; Gavrilovici et al., 2010). The magnitude of these responses increased over the range of 5–100 Hz (5–100 pulses delivered over a period of a second see Figure 1B). However, below 20 Hz (at 5 and 10 Hz) the responses were very small and highly variable. In general, we found that the activation of this neural circuitry was most robust within the range of 20–80 Hz (β–γ range). In Figures 1C–E we show the relationships between the stimulation strength and the change in the magnitude of responses in layer II, Den, and layer III, respectively, over the range of 5–100 Hz. In high β (20 Hz) and continuing into the γ frequency range the input/output relation was non-linear with higher frequencies being relatively more effective. Above 80 Hz (100 Hz) the responses plateaued. For all responses the input/output relation could be fitted with a sigmoidal curve. The midpoint of these curves showed that the half maximal activation for the excitation (layer II) was about 40 Hz while the inhibition of layer III was half maximal at about 20 Hz. The activation of the DEn was essentially the same as the layer II responses although smaller in magnitude. It is important to note that the decrease in activity is not a bleaching artifact as reduced activity and/or hyperpolarization is associated with an increase in dye fluorescence while depolarization causes a decrease in fluorescence.

**Figure 2:**
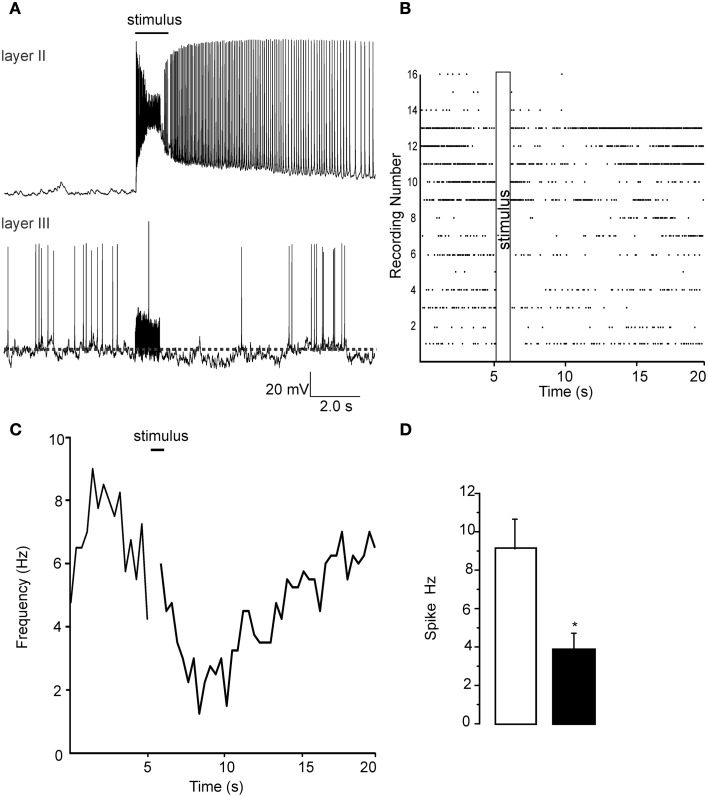
**Patch clamp recordings from Layer II and layer III cells show opposite changes in excitability due to a 1 s long 60 Hz stimulus. (A)** Top trace shows the increase in activity that occurs in layer II pyramidal cells when the LOT is stimulated. Each stimulus pulse causes an EPSP that supports an action potential (not shown). At the end of the stimulus train action potential generation continues for up to 10 s after the stimulus. Lower trace in **(A)** shows a recording from a layer III cell where a reduction in action potential frequency was evident. Note that the stimulus is unable to trigger any kind of response during the train but a small slowly developing hyperpolarization was evident in most recordings. **(B)** A raster plot of the 16 recordings analyzed in these experiments is shows that although there was a wide variability in the initial spike frequency there was a uniform reduction in frequency immediately proceeding the stimulus train. In **(C)** we show the time course of the reduction spike frequency over a 14 s period after the stimulus of LOT. **(D)** Control recordings had a spontaneous frequency of about 8–10 Hz after the stimulus this was reduced to about 4 Hz after the pulse train. ^*^p < 0.01.

To confirm that the signals we observed using the VSDI had a single cell correlate we performed patch clamp recordings from both layer II and layer III cells. We stimulated the LOT (@ 60 Hz) in the same manner as in VSDI recordings. In Figure 2A we show representative recordings, done on separate occasions, showing the excitation of a pyramidal cell while in the layer III recordings the stimulus train resulted in the a reduction of spontaneous firing being reduced. In Figure 2B we show a raster plot of 16 separate recordings showing that after the stimulus train (60 HZ is this example) the spike frequency was reduced. Similarly in Figure 2C a plot of the average frequency of firing was reduced after the train, about 5–10 s after the trains, but was recovered by the end of the sweep (20 s). Figure 2D shows a bar graph where we quantified the average frequency of action potential 5 s before the train and in the 5 s after the trains where the frequency of firing decreased by 62 ± 13% (*n* = 16; *p* < 0.001). Importantly the train did not activate layer III cells, only decreased their firing frequency. The reduction in firing frequency was also usually accompanied by a small hyperpolarization that was difficult to quantify due to the ongoing spontaneous activity. Thus, activation of the LOT decreased the excitability of individual cells located in Layer III, corroborating the VSD recordings.

**Figure 3:**
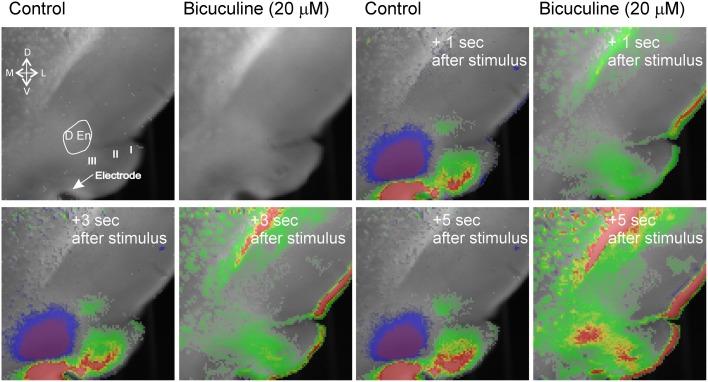
**Blockade of GABA_**A**_ receptor function creates wide spread hyperexcitability of that spreads to adjacent structures.** The blue inhibitory signal is abolished and excitatory activity occurs that spreads into external capsule and striatum. Representative recording from an n of 3 differing slices.

**Figure 4:**
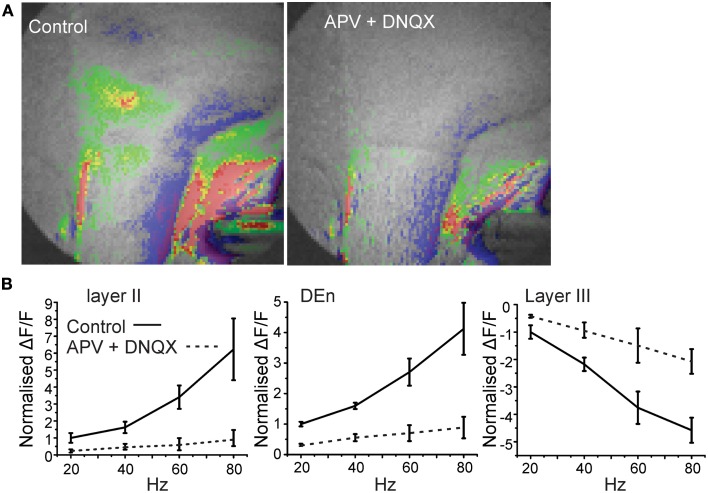
**Blockade of glutamate receptor function significantly attenuates activation of the disinhibitory circuit.** In **(A)** we show a representative image of the response before and after application of 10 μM and 50 μM DNQX and APV, respectively. Image on left was taken after 5 min of perfusion. In **(B)** we show the quantitation of the glutamate receptor blockade over the range of stimulation frequencies used to activate the circuit.

The activity of this circuit responded in a predicable manner. Blockade of GABA_A_ receptor activity by bicuculline (20 μM) abolished all inhibitory responses causing widespread and long lasting excitation of the slice that spread into the external capsule and striatum (Figure 3). Thus, intact inhibitory activity is required to drive the complex activity of this circuit. Likewise incubation of the slices with 10 μM DNQX and 20 μM APV to block excitatory transmission also reduced all activity by about 90% within 5 min (Figure 4).

**Figure 5:**
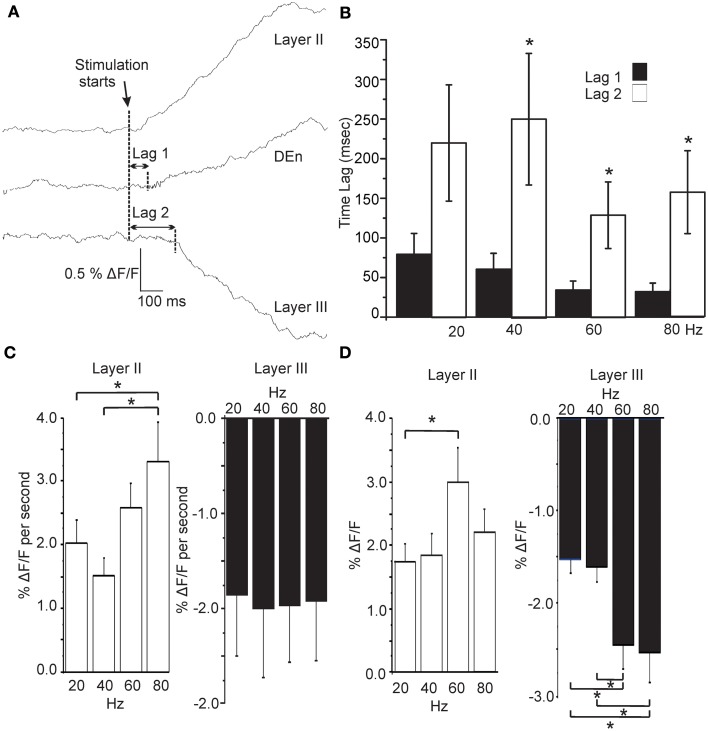
**Activation of each area follows a precise and constant temporal pattern that is independent of stimulation frequency. (A)** Representative traces showing the time lag between activation of Layer II, Den, and Layer III regions (80 Hz stimulation; sample rate 5ms/frame; 200 Hz frames/s). Graph in **(B)** shows that the Lag 1 was consistently shorter than Lag 2. Stimulation frequency had no effect on lag time for either response. **(C)** Effect on the rate of rise of the responses as a function of stimulation intensity. Layer II responses activated more quickly at higher stimulations but Layer III responses did not scale to intensity (DEn responses were the same as Layer II). **(D)** Responses to the same number of pulses at differing stimulation frequencies showed that Layer II responses were most sensitive to a 60 Hz train. Similarly higher frequencies trains were more effective in evoking Layer III responses. (Again DEn responses were the same as Layer II). ^*^p < 0.01.

In Figure 5A we show that the (de)activation of Layer II, the Den, and layer III were not simultaneous and always followed the same temporal sequence. After the initial rise in the response of layer II it was followed by the activation of the DEn (about 50 ms later), which preceded the deactivation observed in layer III (about 200 ms after the layer II response). This occurred at all frequencies between 20 and 80 Hz. There was no effect of the stimulation frequency on the lag times although there was a small trend to shorten lag 1 as the stimulation intensity increased (Figure 5B). However, we found that the activation rate of layer II and DEn responses increased with the number of pulses delivered during the 1 s train, but this was not the case for inhibition produced in layer III (Figure 5C). We also determined whether the responses were sensitive to the frequency of stimulation. Thus, we stimulated the LOT while keeping the number of pulses constant (60), but varied the frequency at which they were delivered. In Figure 5D we show that the 60 Hz stimulus (of those tested) was the most effective at evoking the largest magnitude response in the layer II cells. Similarly, the inhibitory responses were also most sensitive to the higher frequencies. These data show that the temporal activation is not dependent on the number pulses within a 1 s train but is sensitive to the frequency at which they are delivered. Activation is likely limited by conduction velocity of the neurons being activated and by synaptic delay(s) as well as the summation of synaptic potentials that each pulse delivers (see ensuing discussion section). So while the number of pulses did not affect these attributes, the stimulation frequency (same number of pulses) was able to scale the rate of activation and magnitude of the responses.

**Figure 6:**
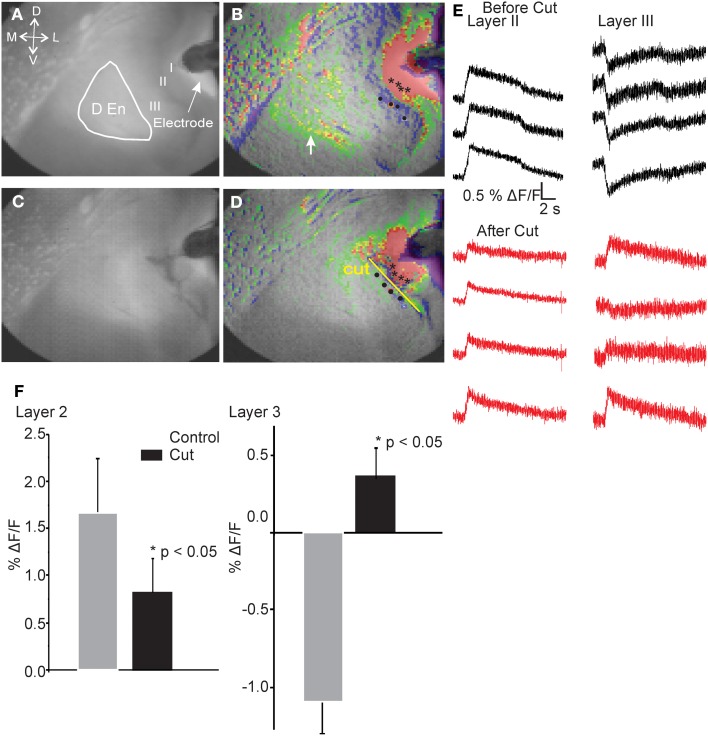
**Cutting the connections between Layer II and deeper layers of the PCtx abolished activation of the DEn and converted the inhibition in Layer III to excitation.** Representative images before **(A,B)** and after **(C,D)** cutting the slice. Black asterisks indicate spots where representative traces were taken. Yellow Line shows the pixels that were used to follow and quantify the response before and after introducing the cuts. Identical regions were quantified. In **(E)** we show that the reduced excitation of layer II along four pixels while traces in layer III are inverted and became excitatory. Responses in the DEn were completely abolished. In **(F)** we show the quantitation of the reduction of excitation of layer II and the conversion of layer III responses to excitatory ones.

Our interpretation of these observations led us to postulate that there is a neural circuit that functions through a feed forward disinhibitory loop where the initial activation of the layer II cells excites inhibitory cells in the DEn that, in turn, inhibit the interneurons in layer III. This “shutting off” of the inhibition in layer III then augments the efficacy of the ongoing stimulus of the LOT. This hypothesis is also based on the knowledge that both the PCtx and the DEn nuclei are highly interconnected, with the DEn having many inhibitory cells whose axons penetrate into layer III. As well, pyramidal cells of layer II have strong excitatory connections into the DEn (Haberly and Presto, 1986). Our hypothesis predicts that interrupting the conduction of the axons that emerge from the cell bodies of the layer II pyramidal cells and penetrate into layer III and DEn should attenuate the activation DEn and therefore, the inhibition of the layer III. As no pharmacological treatment could accomplish this manipulation we choose to cut the brain slice just medial to the layer II to see whether this manipulation would block DEn activation and attenuate the disinhibition. To do this we first recorded control responses. We then carefully cut the slice just below layer II and then stimulated again. Figure 6 shows that after the cut, the activation of the DEn was completely abolished and the disinhibition in Layer III was converted to excitation (presumably from the feed forward activation of layer III interneurons that is also known to function in this circuit; Suzuki and Bekkers, 2010). These data show that activity originating from pyramidal cell layer is required to activate the DEn. Without this activation the disinhibitory phase is not observed.

**Figure 7:**
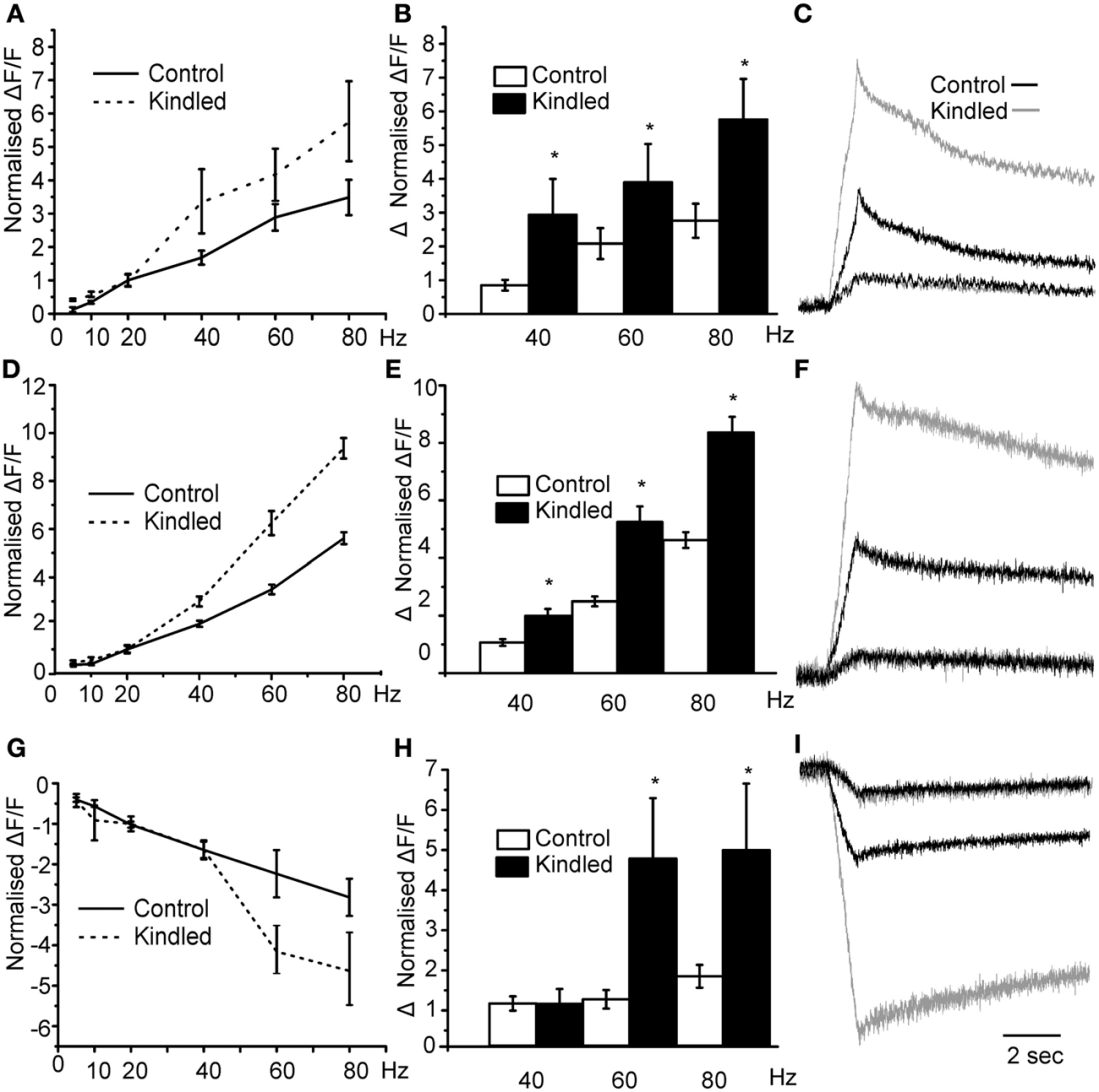
**Epileptic seizures augment the responses to stimulation at the frequencies between 20 and 80 Hz for all areas.** In panel **(A)** we show that responses to stimulation of control vs. kindled slices. Kindled slices produced more robust responses increasing up to 6-fold in comparison to controls. In **(B)** we show average difference between the normalized response (20 Hz) and the higher frequency ones. Kindled brain caused larger responses than those produced in control brain slices (^*^indicates *p* < 0.05 in comparison to control differences). In **(C)** we show representative traces of Control (black trace) and kindled (gray trace) scaled so that the 20 Hz responses are the same. At 80 Hz the response in the kindled brain was about 6 times bigger than the 20 Hz response while the Control responses were only about 3.5 times bigger. In Panels **(D–F)** we compare the stimulation intensity vs. the normalized fold change for DEn. Again after kindling the responses are significantly bigger than in controls. Panel **(F)** compares responses at 80 HZ where DEn response were up to 10 times the 20 Hz response compared to about 5-fold in controls. In Panel **(G)** we show that disinhibition of the layer III is higher in kindled slices at 60 and 80 Hz stimulations than in controls. In panel **(H)** we show that changes were larger, with the 80 Hz hyperpolarizing response about 4.5-fold bigger than the 20 Hz responses. In control slices is only about 2.5 times bigger. In panel **(I)** we show the representative traces of Control and kindle slice responses in Layer III.

To test our hypothesis by another manipulation we used brain slices from rats in which seizures are produced by stimulation of electrodes implanted in the basal lateral amygdala, a process known as kindling. After kindling we have previously shown that the miniature postsynaptic inhibitory currents on interneurons of the PCtx were larger in amplitude and long lasting resulting an increased in the current density of about 100% (Gavrilovici et al., 2006). Our hypothesis in this case was that stimulating the LOT should produce larger responses in the layer II since in the kindled brain the magnitude of the disinhibition should be larger. We found that the threshold for activation of this network response was reduced in kindled slices compared to controls (Sham 15 ± 3 μA vs. 7 ± 2 μA *p* < 0.05). Thus, as would be expected, the excitability of the circuit is increased after kindling. In Figure 7A we show that, in kindled brain slices, the magnitude of the normalized layer II responses was larger than those produced in control slices. Specifically, we show that in control slices the magnitude of the response increased by about 3 fold at the maximum stimulation intensity of 80 Hz. However, in the kindled slices the magnitude of activation was greater in kindled slices. This is quantified in Figure 7B where we show that the average difference in the magnitude of the 40, 60, and 80 Hz responses and the 20 Hz response were greater in the kindled slices. In Figure 7C we show traces where the 20 Hz responses in kindled and control slices have been scaled to have the same magnitude and the 80 Hz response from the same recording is compared. The relative change in the responses is much greater in the kindled slice than in controls. Similarly, the magnitude of D En responses was greater in kindled slices than in controls (Figures 7D–F). Although there was no difference up to 40 Hz in the inhibitory responses between controls and kindled we found that the 60 and 80 Hz responses increased more dramatically in the kindled slices (Figures 7G,H). In Figure 7I we show the inhibitory response in control and kindled slice scaled according to the 20 Hz response. Thus, there is more disinhibition of Layer III after kindling. These data show that after kindling the disinhibition of layer III is increased with a concomitant increase in layer II excitability. This may occur through the increased activation of the DEn which in turn inhibits more cells in Layer III and/or the increased inhibitory current density we have previously reported (Gavrilovici et al., 2006).

## Discussion

**Figure 8:**
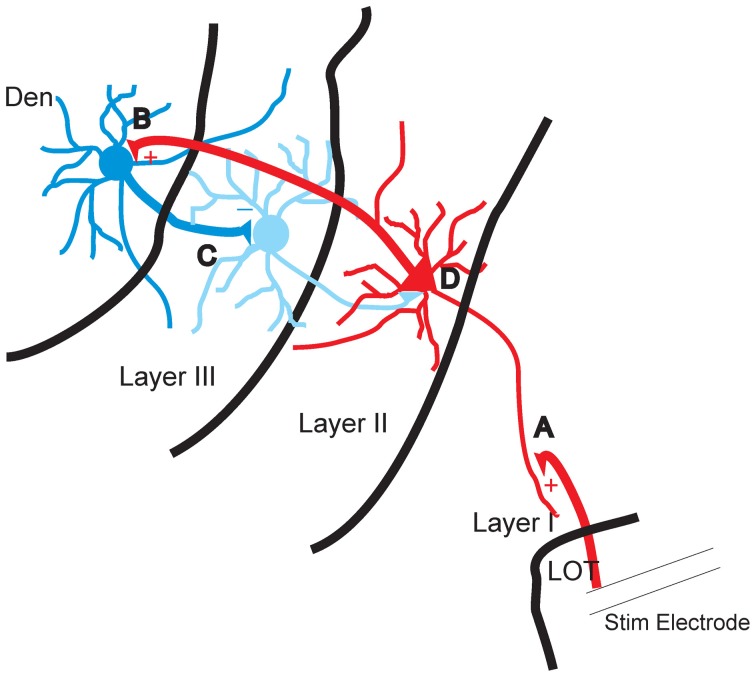
**Diagram of proposed feed forward disinhibitory circuit in piriform cortex. (A)** The apical dendrites of the pyramidal cells in layer II are excited by stimulation of the lateral olfactory tract (LOT). **(B)** The axons of layer II pyramidal cells augment the activity of interneurons of DEn. **(C)** Excited interneurons of DEn inhibit the activity of layer III interneurons. **(D)** Disinhibition of interneurons of layer III that innervate layer II can no longer attenuate the activity of the pyramidal cells.

Disinhibition for over 50 years has been postulated to be a potential mechanism that can increase the excitability of neural circuits yet to our knowledge there are very few demonstrations where this has been shown to occur. The data presented here show that the inhibition of layer III, which is almost entirely composed of GABAergic interneurons, coincides with the development of excitation of the layer II which is largely composed of excitatory pyramidal cells (Gavrilovici et al., 2010, 2012). Disrupting the flow of activity from layer II abolished the inhibition of layer III while reducing layer II activation. This feedback disinhibition seems to be mediated by the DEn as cutting axons that connect it to layer II prevented its activation and the subsequent induction of disinhibition. Our observations are also supported by ample anatomical evidence that there are wide spread reciprocal connections between these areas. Axons from the PCtx innervate the DEn and GABAergic axons originating in the DEn innervate the PCtx, particularly layer III (Haberly et al., 1987; Behan and Haberly, 1999) see Figure 8 for diagram.

We have previously shown that after kindling inhibitory current density on layer III interneurons is increased (Gavrilovici et al., 2006; Kia et al., 2011). We suggested that this effect may be partially responsible for seizure activity as the inhibitory neurons would be less excitable. We have also shown recently that interneuron excitability is reduced due to the increased expression of a potassium channel (Gavrilovici et al., 2012); Kv 1.6. We now show that the activation of the DEn is augmented after kindling (Figures 7D–F). The overall effect of these changes will all contribute to the reduced activity of layer III. Thus, there appears to be a confluence of mechanisms that all tend to increase excitability of the pyramidal cell layer. Overall, our data suggest an unrecognized and complex neural mechanism, feed forward disinhibition, which may subserve seizure activity. Furthermore, our observations may explain why the PCtx and DEn are the most seizurogenic regions of brain (Piredda and Gale, 1985; Demir et al., 1999, 2000, 2001). Indeed, the DEn was recognized over 30 years ago to readily support convulsive and/or seizure activity and was termed as the area *tempestus* (Piredda and Gale, 1985). Injections of convulsants into the DEn caused epileptiform activity while injections of GABA_A_ receptor activators suppressed epileptiform bursts. Electrophysiological evidence that the epileptiform activity is easily supported in this region has been demonstrated in another study, but the interpretation of how this may occur did not point to this type of mechanism. (Hoffman and Haberly, 1996) recognized that epileptiform activity originated in the endopiriform nucleus but interpreted their results as being due to the reduction of feedback inhibition. Our data point to the interpretation that the high excitability of this region is due to augmentation of a feedback disinhibitory loop.

These observations were made by using a VSDI a technique that is able to “view” the activity of a brain slice with high spatial and temporal resolution. VSDI has been used in other brain regions to follow activity where both excitatory and inhibitory activity was observed (Carlson et al., 2011; Foust et al., 2011). The signals that we observed are most likely an average of the membrane potential both superficially and deeper into the slice. This would also be anatomically heterogeneous, as well, with both cell bodies and the neuropil all contributing to the signal. Thus, the depolarizing and hyperpolarizing signals are indicative of membrane potential changes that are an average of the ongoing activity both sub and super threshold on the surface and to some degree in the deeper layers of the slice. However, action potentials, owing to their large magnitude in comparison to other sub-threshold voltage fluctuations, likely account for most of the signal. Our patch clamp recordings confirm that LOT stimulation does indeed increase the activity of the layer II cells with a concomitant reduction of action potential generation in layer III neurons. These latter responses were independent of any preceding activation of the cells during the stimulus train. So the reduced action potential generation does not appear to be associated with any local feedback inhibition arising within layer III. These points are important as they support the idea that the suppression neural activity is due to activity from cells (we contend layer II and DEn cells) that are not within layer III. One important anomaly in our data however, is the relative long lag times we have shown to occur during activation of this circuit. For a tri-synaptic pathway a 200 ms lag time is an order of magnitude slower than one might expect. The simplest explanation is that the long lag time reflects the time required for synaptic activation to have an effect on the action potential firing which we have indicated probably dominates the VSDI signal. Although the recordings from layer II pyramidal neurons seem to match the time course of the VSDI signal the peak of the suppression of the action firing in the layer III occurs about 2–3 s after the end of the stimulus train (see Figure 2C). This is an important discrepancy that is difficult to reconcile. However, we did observe that during the stimulus train there were few action potentials thus, the onset of the stimulus suppresses the spontaneous firing relatively quickly so in this respect there is good agreement between the two kinds of signals. Perhaps the best explanation is that the VSDI signal represents a response that summates activity “through” a number of tissue layers that are both in and out of focus in a brain region that is not as uniform as the densely packed layer II and therefore, exact temporal concordance may not occur.

The potential role of disinhibition as a mechanism that could drive or potentiate excitation is largely accepted. Indeed the recognition that the inhibition of connected interneurons networks is important for gating pyramidal cells networks oscillations could be viewed as a form of disinhibition. However, very few studies have shown that inhibition of an interneuron population augmented principal cell excitability. The only study that we are aware, that was just recently reported, has shown that the inhibition of somatostatin positive interneurons (SOM-IN) in sensory cortex increased the firing of pyramidal cells in layers 2/3 but decreased the firing of layer 4 principal neurons. The authors suggested that these observations are consistent with the idea that the decreased activity of the SOM-IN must innervate another IN population that normally reduces Layer 4 activity (Xu et al., 2013). Another similar mechanism has been reported in which a form of auto inhibition occurs, in rat olfactory bulb, whereby the release of GABA from an individual cell activates GABA_A_ receptors on the same neuron which then in turn impacts the excitability of projection neurons (Smith and Jahr, 2002). Both these observations are similar to the ones we have provided here except that we show that activation of the DEn coincides with the inhibition of layer III interneurons that target the layer II principal cells of the PCtx. However, the observation that an entire subfield of one brain region is effectively “taken off line” altering the gain of an ongoing excitatory stimulus has not been reported, to our knowledge. The disinhibition reported here is also selective to the frequency of stimuli since low frequency stimulation was unable to trigger the potentiation. The net result, presumably, is to tune the response of the PCtx to the high frequency action potential volleys that can originate in the olfactory bulb. It is also important to note that the activation of this circuit occurs in a frequency range of stimulation (β–γ range) which is known to be a mode of communication between olfactory bulb and PCtx (as well other lower frequency ones; Kay, 2003; Neville and Haberly, 2003; Ravel et al., 2003; Lagier et al., 2004; Zibman et al., 2011). Interestingly this increase in efficacy may come at a “high cost” as this region of the brain also easily supports epileptic seizures.

VSDI imaging has been used on other studies to examine epileptiform activity. Many of these studies have been from *in vitro* hippocampal slice preparations (Dasheiff and Sacks, 1992; Jackson and Scharfman, 1996; Demir et al., 2000; Ang et al., 2006; Carlson and Coulter, 2008; Kibler and Durand, 2011; Coulter et al., 2011). The main findings of these studies showed numerous spatial and temporal sources of activity that would otherwise be difficult to identify with conventional electrophysiological techniques. For example, Chang et al. (2007) identified a region in the CA1 near the CA2 that more readily supported epileptiform bursts and theta burst induced LTP. These observations implied that the intrinsic “wiring” of this region is uniquely sensitive to heightened activity. In another study seizure generation was found to occur in two differing regions of the hippocampus that seemed to interact in manner that increased the likelihood of the propagation of seizure activity throughout the hippocampus. The authors suggested that these multisite origins of the seizures behaved as coupled oscillators that drove the epileptiform activity (Derchansky et al., 2006). Importantly, these data suggest that epileptic foci may not be as discreet anatomically as is assumed. In another study, VSDI revealed that the emergence of the hyperexcitability “transforms” lamellar spread of activity to one where the excitability abnormally propagates orthogonally through the entire hippocampus (Kibler and Durand, 2011). Finally VSDI imaging has also indicated that seizure propagation is not dependent on intact synaptic connections since epileptiform waves of excitation can spread across mechanical lesions. These observations implicate increases in extracellular potassium as being sufficient to synchronize neuron activity across relatively large regions of the hippocampus (Lian et al., 2001). Overall these studies have shown numerous mechanisms within the hippocampus that can support seizure activity.

In summary, our observations provide new evidence of a neural circuit that when activated augments the efficacy of the initial excitatory input by disinhibition. Our data also provide the basis to explore an entirely new line of questioning about the mechanisms by which epileptic seizures may occur. Indeed, this type of circuitry may be important in many regions of the brain where the gain of control of synaptic excitation may be governed depending on the frequency of the excitatory stimuli.

### Conflict of interest statement

The authors declare that the research was conducted in the absence of any commercial or financial relationships that could be construed as a potential conflict of interest.
