# Corrigendum: Gain control of gamma frequency activation by a novel feed forward disinhibitory loop: implications for normal and epileptic neural activity

**DOI:** 10.3389/fncir.2014.00070

**Published:** 2014-08-21

**Authors:** Zeinab Birjandian, Chakravarthi Narla, Michael O. Poulter

**Affiliations:** Molecular Medicine Research Group, Department of Physiology and Pharmacology, Robarts Research Institute, The University of Western OntarioLondon, ON, Canada

**Keywords:** epilepsy, disinhibition, interneuron, endopiriform cortex, olfactory cortex, piriform cortex

Figure 1 of the article by Birjandian et al. (2013) contained a minor error in the legend, which we hereby rectify. In the original figure legend panel **(C)** is described as two examples of whole cell patch recordings made from layer II and layer III neurons. But in fact panel **(C)** correspond to normalized change in ΔF/F vs. stimulus intensity from 8 recordings in the layer II. Panels **(D,E)** correspond to normalized change in ΔF/F vs. stimulus intensity from 8 recordings in DEn and Layer III but not layer II. We therefore would like to make a correction to Figures 1C–E.

## Conflict of interest statement

The authors declare that the research was conducted in the absence of any commercial or financial relationships that could be construed as a potential conflict of interest.
